# Evolutionary and expression analyses of soybean basic Leucine zipper transcription factor family

**DOI:** 10.1186/s12864-018-4511-6

**Published:** 2018-02-22

**Authors:** Man Zhang, Yanhui Liu, Hang Shi, Mingliang Guo, Mengnan Chai, Qing He, Maokai Yan, Du Cao, Lihua Zhao, Hanyang Cai, Yuan Qin

**Affiliations:** State Key Laboratory of Ecological Pest Control for Fujian and Taiwan Crops; Fujian Provincial Key Laboratory of Haixia Applied Plant Systems Biology, Center for Genomics and Biotechnology, College of Plant Protection, College of life science, College of crop science, Fujian Agriculture and Forestry University, Fuzhou, 350002 Fujian Province China

**Keywords:** Soybean, *bZIP* genes, Phylogenetic analysis, Expression, Synteny analysis, Water stress

## Abstract

**Background:**

Soybean, a major legume crop native to East Asia, presents a wealth of resources for utilization. The basic leucine zipper (bZIP) transcription factors play important roles in various biological processes including developmental regulation and responses to environmental stress stimuli. Currently, little information is available regarding the bZIP family in the legume crop soybean.

**Results:**

Using a genome-wide domain analysis, we identified 160 *GmbZIP* genes in soybean genome, named from *GmbZIP1* to *GmbZIP160*. These 160*GmbZIP* genes, distributed unevenly across 20 chromosomes, were grouped into 12 subfamilies based on phylogenetic analysis. Gene structure and conserved motif analyses showed that *GmbZIP* within the same subfamily shared similar intron-exon organizations and motif composition. Syntenic and phylogenetic analyses identified 40 *Arabidopsis bZIP* genes and 83 soybean *bZIP* genes as orthologs. By investigating the expression profiling of *GmbZIP* in different tissues and under drought and flooding stresses, we showed that a majority of *GmbZIP* (83.44%) exhibited transcript abundance in all examined tissues and 75.6% displayed transcript changes after drought and flooding treatment, suggesting that *GmbZIP* may play a broad role in soybean development and response to water stress.

**Conclusions:**

One hundred sixty *GmbZIP* genes were identified in soybean genome. Our results provide insights for the evolutionary history of bZIP family in soybean and shed light on future studies on the function of *bZIP* genes in response to water stress in soybean.

**Electronic supplementary material:**

The online version of this article (10.1186/s12864-018-4511-6) contains supplementary material, which is available to authorized users.

## Background

Plants are exposed to extreme environment during their life cycle. Growth and development would be delayed once plants are exposed to terrible environmental conditions, such as drought, cold, high salt, and heat. To survive these stress conditions, plants regulate the expression of stress related genes. Transcription factors (TFs) play key roles in regulating the expression of functional proteins when plants encounter extreme environment conditions. Among TF families, the basic leucine zipper (bZIP) transcription factor family is a kind of conserved family that is prevalent in plants, animals, and microorganism. They possess a conserved 40–80 amino acid bZIP domain that has two structural features: a basic DNA binding region and a leucine zipper dimerization motif [1, 2]. The basic region is conserved, containing an invariant motif N-× 7-R/K-× 9 with about 18 amino acid residues [3–5]. The leucine zipper has a less conserved dimerization motif composing a heptad repeat of leucine residues, or other bulky hydrophobic amino acids that are involved in recognition and dimerization [4–6].

Considerable evidence has shown that bZIP TFs take part in different biological processes. *OsABF1* (*Oryza sativa* ABA responsive element binding factor 1) can directly regulate and suppress *Ehd1* expression and confer a later flowering phenotype in rice [7]. *OsABF2*, as a transcriptional regulator, can modulate the expression of abiotic stress-responsive genes through an ABA-dependent pathway [8]. *OsbZIP23* transcription factor acts as a central regulator in ABA signaling and biosynthesis, and overexpressed *OsbZIP23* shows significantly improved drought and salt tolerance, and BiFC assay in rice protoplast indicated that *OsbZIP23* could interact with a *SnRK2* protein kinase (SAPK2) [9]. *ZmFEA4*, an ortholog of *Arabidopsis* PERIANTHIA bZIP transcription factor in maize, promotes differentiation in the meristem periphery by regulating auxin-based responses [10]. The *Arabidopsis* transcription factor *AtbZIP53* regulates seed maturation by influencing heterodimerization and protein complex formation [11]. *AtbZIP11* activates auxin-mediated transcription by recruiting the histone acetylation machinery [12]. The bZIP TFs also play key roles in regulation of plants response to various biotic and abiotic stresses, such as glucose-ABA signaling [13], sugar signaling during metabolism [14], ABA signaling for osmotic stress responses during vegetative growth [15], seed germination and flowering time [16], response to zinc deficiency [17], lipid stress responses [18], floral patterning [19], and auxin- mediated histone acetylation [20].

Due to their crucial role as regulator of responding to biotic and abiotic stresses, bZIP transcription factor families have been identified in various plant species. Based on their DNA binding specificity and amino acid sequences in basic and hinge regions, 89bZIPs were identified in rice [21]; 64 were found in cucumber based on predicted structural features [22], 92 in sorghum through genome-wide identification and characterization [23], 136 in *Brassica rapa* according to the presence of the UARR and LCRs [24]. Soybean, a major crop, plays an important role in food and industrial production. However, the genome-wide characterization of bZIP transcription factors in soybean has not yet been carried out. Here, we identified 160 *bZIP* genes from soybean genome and conducted a comprehensive genome-wide analysis of bZIP transcription factors in soybean in comparison with *Arabidopsis*. To infer the potential functions of these genes, the expression profile of *bZIP* genes in different soybean tissues and their response to drought and flooding were analyzed.

## Methods

### Identification of *bZIP* gene family in soybean and *Arabidopsis*

The whole bZIP protein sequences from soybean and *Arabidopsis* were obtained from Phytozome12 (https://phytozome.jgi.doe.gov/pz/portal.html), downloading the HMM profiles of the bZIP domain (PF00170, PF07716, PF03131) (http://pfam.sanger.ac.uk/) as queries to search predicted bZIP proteins in the soybean dataset using HMMER software 3.0 (HMMER: http://hmmer.wustl.edu/) with a threshold of e-value < e^− 5^ [25]. Subsequently, BLAST searches were performed to identify the predicted bZIPs in soybean database with all the *Arabidopsis* bZIPs as queries. Finally, candidate genes were examined to confirm the protein sequences derived from the selected soybean bZIPs, using the domain analysis programs of Pfam (Protein family: http://pfam.sanger.ac.uk/) and SMART (Simple Modular Architecture Research Tool: http://smart.embl-heidelberg.de/).

### Phylogenetic analysis

Multiple sequence alignments of soybean and *Arabidopsis* bZIP were performed using muscle (http://www.ebi.ac.uk/Tools/msa/muscle/). Phylogenetic and molecular evolutionary analyses were generated using MEGA 6.0 software (http://www.megasoftware.net) and the FastTree 2.1.7 software (http://www.microbesonline.org/fasttree/).

### Protein properties and sequence analyses

The molecular weight and isoelectric points of predicted GmbZIP proteins were detected using the ExPASy proteomics server (http://expasy.org/) [26]. The MEME program (http://meme.nbcr. net/meme/cgi-bin/meme.cgi) was employed to identify the conserved motifs in full-length soybean bZIP proteins with the following parameters: maximum number of motifs was 20 and the optimum width of motifs was set between 10 and 50 [27]. The *GmbZIP* gene structure analysis was displayed with GSDS (http://gsds.cbi.pku.edu.cn/).

### Synteny analysis and chromosome localization

We used BLASTP to search for potential anchors (E < 1e-5, top 3 matches) within the soybean genome and between the soybean and *Arabidopsis* genomes and those containing soybean and *Arabidopsis bZIP* genes were identified and analyzed. All data used to analyze the expansion patterns of the *GmbZIP* family were shown in Additional file 1: Table S3 and Additional file 2: Table S4. Diagrams were generated using the Circos program (version 0.69) (http://circos.ca/). The synonymous (Ks) and non-synonymous (Ka) nucleotide substitutions between orthologous gene pairs were calculated based on the comparative synteny map between the soybean and Arabidopsis genomes, using Clustal W [28], PAL2NAL [29] and yn00 program of the bio-pipeline (https://github.com/ tanghaibao/ bio- pipeli ne).

### Expression analysis of *GmbZIP* genes in different tissues

Transcriptome analysis was performed to identify expression patterns in representative tissues, including roots, root hairs, stems, nodules, leaves, shoot apical meristem, flowers, pods, and seeds. Transcriptome data of soybean tissues in different stages was obtained from Phytozome12 (https://phytozome.jgi.doe.gov/pz/portal.html) (Additional file 3: Table S5). Finally, heatmap of *GmbZIP* expression profile was produced by the pheatmap packages in R.

### Plant material and treatments

The soybean seeds were obtained from center for genomics and biotechnology in Fujian Agriculture and Forestry University (FAFU), and were grown in a greenhouse in the pots containing nutritional soil. Two-week-old soybean seedlings were transferred to the drought and flooding conditions for a week, and drought stress was imposed by withholding water for 7 days, and flooding stress was imposed by placing the pots into a bigger pot with a trashcan liner filled up to a water level of 4 cm above the soil surface for 7 days. After 7 days of treatment, all the leaves were sampled for experiment [30].

### Quantitative real-time PCR

Total RNA was extracted from the soybean leaves under the stress-treatment and unstressed using an RNA plant extraction kit (OMEGA, China) according to the manufacture’s instruction, and approximately 1 μg of purified total RNA was reverse transcribed to cDNA in a 20 μL reaction volume using AMV reverse transcriptase (Takara) according to the supplier’s instructions. To analyze the relative transcript levels of selected genes, real-time PCR was performed with specific primers according to the Bio-Rad Real-time PCR system (Foster city, CA, USA) and the SYBR Premix Ex II system (TakaRa Perfect Real Time). The qRT-PCR program was: 95 °C for 30 s;40 cycles of 95 °C for 5 s and 60 °C for 34 s; 95 °C for 15 s. The primers used for RT-PCR are listed in Additional file 4: Table S9.In each case, three technical replicates were performed for each of at least three independent biological replicates [31]. Quantities of standard RNA were prepared by diluting cDNA (1, 10^− 1^, 10^− 2^, 10^− 3^, 10^− 4^, and 10^− 5^) and only Ct values less than 40 were used to calculate correlation coefficients (R^2^ values) and amplification efficiencies (E) from the slope generated in Microsoft Excel 2013, based on the eq. E = [10^−(1/slope)^ − 1] × 100%. All PCR assays showed efficiency values between 95% and 110% (Additional file 4: Table S9) [32].

## Results

### Identification of the soybean bZIP gene family

**Fig. 1:**
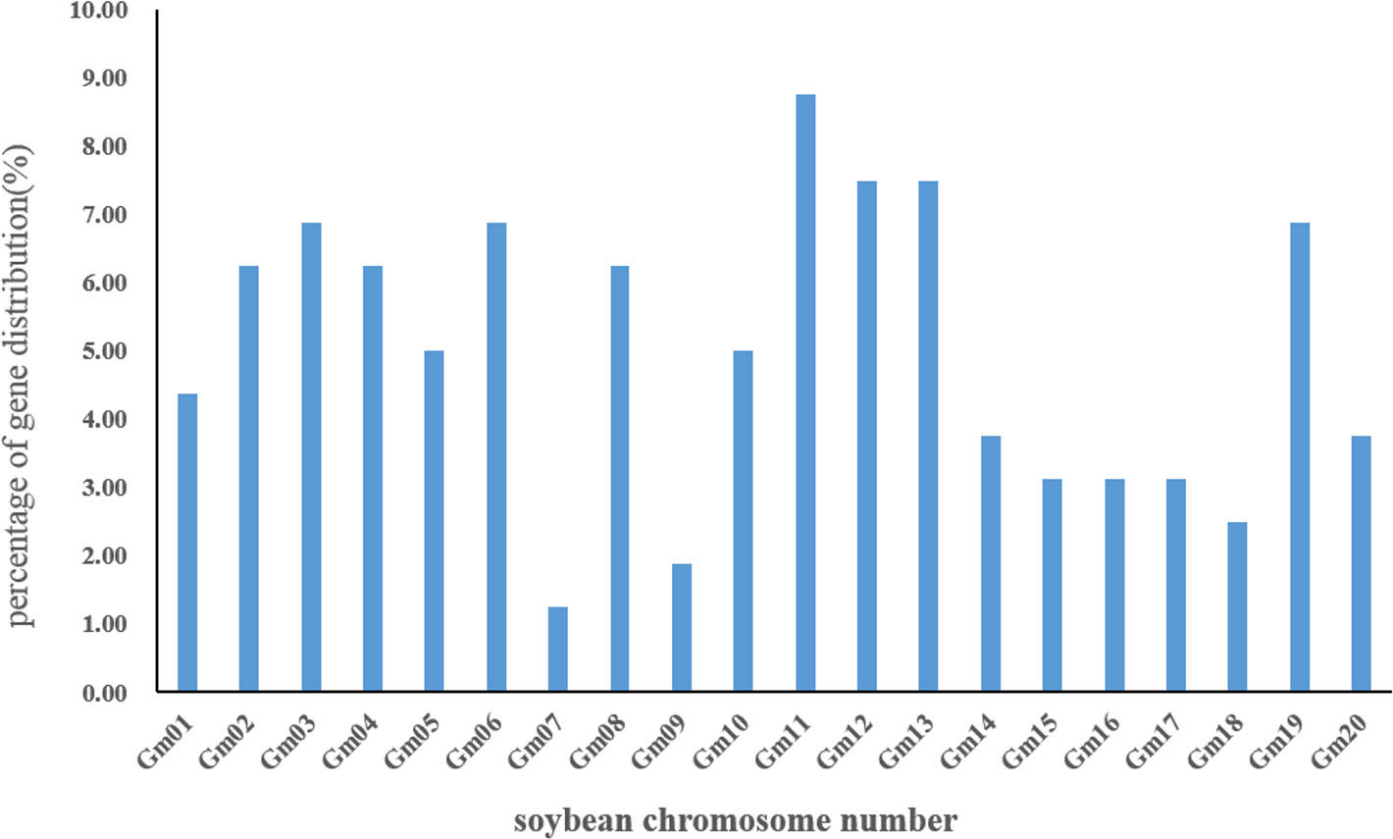
The distribution of *bZIP* genes on soybean chromosomes, shown as percentages

To identify *GmbZIP* genes, BLAST and Hidden Markov Model searches were used to search the soybean genome database with bZIP sequences from *Arabidopsis* as query. A total of 160*GmbZIP* genes were identified from soybean genome, named *GmbZIP1* to *GmbZIP160*. The 160*GmbZIP* genes were unevenly distributed among 20 soybean chromosomes, with chromosome 11 containing the most (8.75%), followed by chromosome 12 and 13 with about 7.5%, chromosomes 7 and 9 less than 2% (Fig. 1). The full-length of 160 GmbZIP proteins varied from 95 (GmbZIP52) to 853 (GmbZIP44) amino acid residues with CDS ranging from 288 to 2562 bp, relative molecular mass distributing from 10.93 (GmbZIP95) to 157.28 (GmbZIP47) kDa, following with isoelectric points ranged from 4.76 to 10.63 (Additional file 5: Table S1; Additional file 6: Table S2).

### Expansion patterns of the soybean bZIP family

**Fig. 2:**
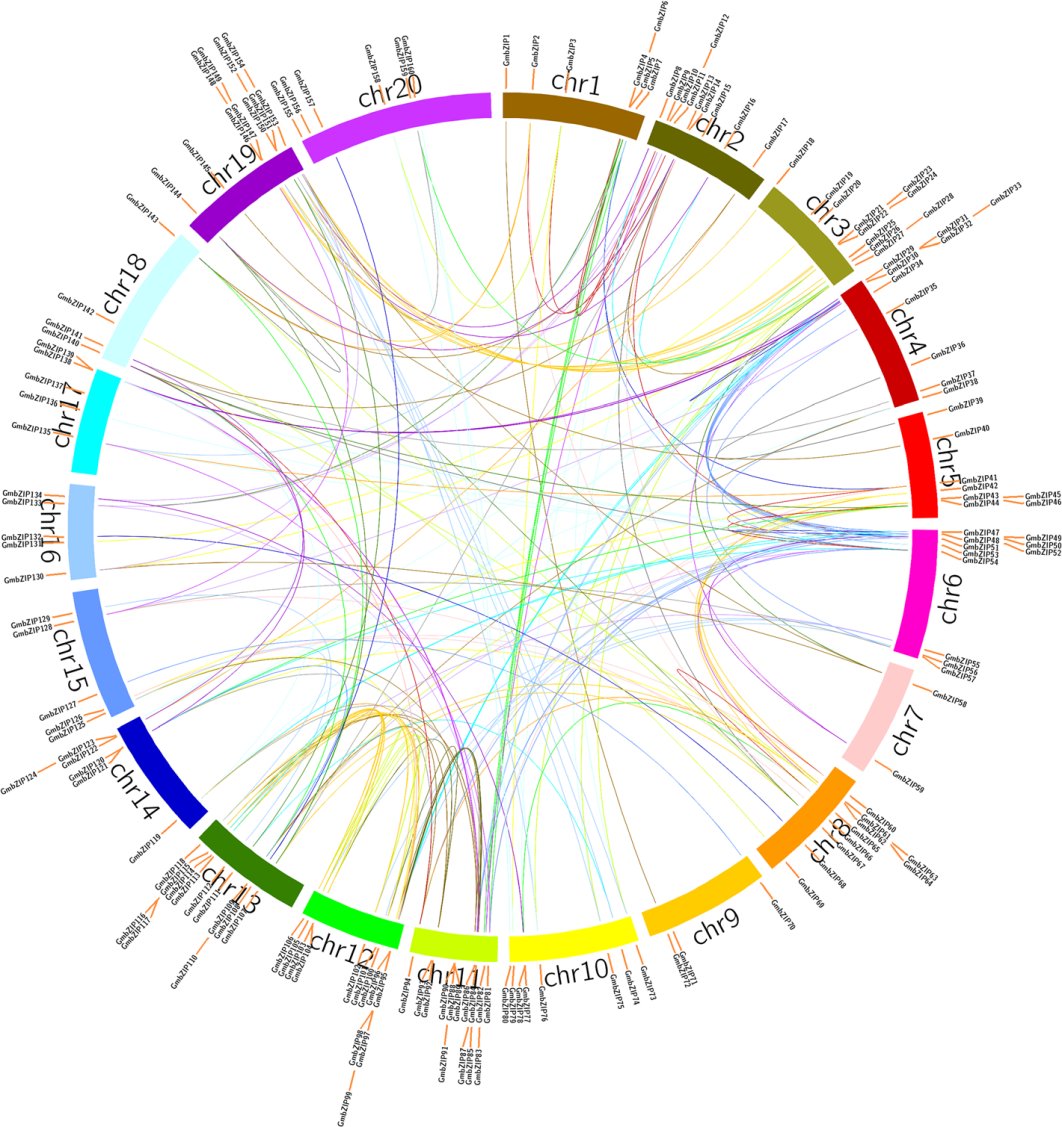
Distribution and synteny analysis of soybean *bZIP* genes. In the picture, the 20 soybean chromosomes are represented in different color partial circles and Chromosome numbers are indicated at the top of each bar. *GmbZIP* genes in different chromosomes are indicated by vertical red lines. Colored bars instruct bZIP syntenic regions on the soybean *bZIP* gene family

**Fig. 3:**
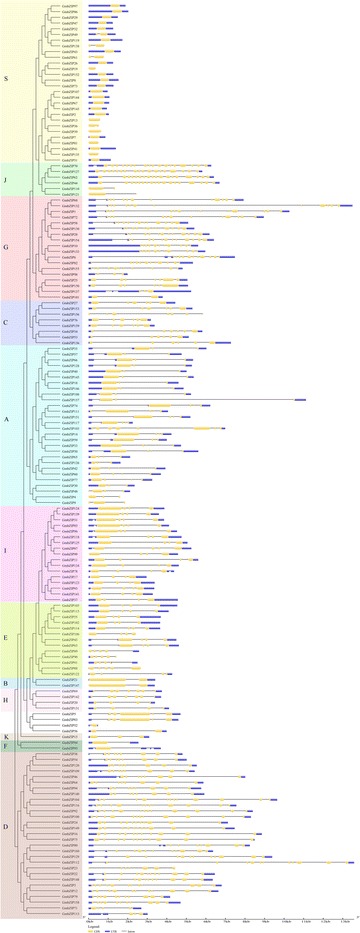
The exon-intron structure of *GmbZIP* genes based on the evolutionary relationship. The evolutionary tree was carried out with FastTree. Exon-intron analyses of *GmbZIP* genes were created with GSDS. Lengths of introns and exons of *GmbZIP* genes were exhibited proportionally

Segmental and tandem duplications provide two important mechanisms for the expansion of gene families and evidence of both could be found in an analysis of the GmbZIP gene family [33]. As displayed in Fig. 2, some *GmbZIP* gene pairs were distributed close to each other on the chromosomes, whose gene structure and coding sequence show high similarity (Fig. 3); for example, *GmbZIP47* and *GmbZIP49* on chromosome 6,*GmbZIP114*and *GmbZIP115* on chromosome13.The segmental duplication blocks among genome were compared to the chromosomal location of all the *GmbZIP* genes. In total, more than 190 pairs of *GmbZIP* genes, such as *GmbZIP1*/*GmbZIP72*, *GmbZIP24*/*GmbZIP75*, *GmbZIP153*/*GmbZIP159*, as well as others (Additional file 1: Table S3), were located at two different chromosomes. In general, these results showed that the expansion of *GmbZIP* genes might result from the tandem and segmental duplications among these genes.

### Gene structures and phylogenetic relationships of *GmbZIP* genes

**Fig. 4:**
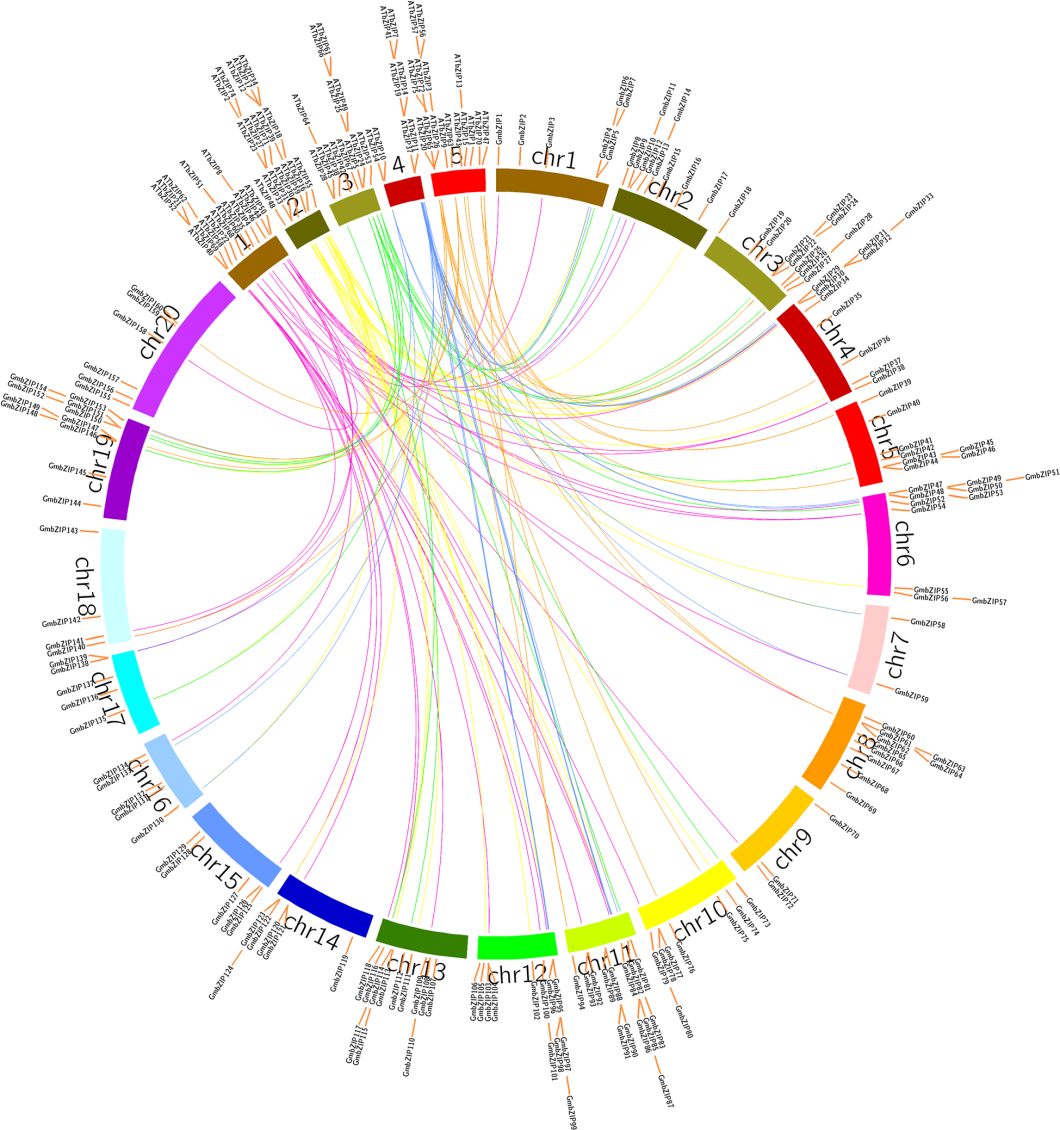
Synteny analysis of *bZIP* genes between soybean and *Arabidopsis*. In the picture, the 20 soybean chromosomes are represented in different color partial circles and Chromosome numbers are indicated at the top of each bar. Soybean and *Arabidopsis* bZIP genes are instructed by vertical red lines. Colored lines connecting two chromosomal regions indicate syntenic regions between soybean and *Arabidopsis* chromosomes

To analyze the evolutionary history of the *GmbZIP* gene family, an unrooted phylogenetic tree was generated by a multiple sequence alignment of predicted GmbZIP proteins (Fig. 3). The result showed that bZIP proteins from the same group tended to cluster together, which is consistent with the two-species analysis (Fig. 4). To further understand the evolutionary relationships among *GmbZIP* genes, we then analyzed intron-exon organizations. As shown in Fig. 3, 25 *bZIP* genes have no intron, all of which belong to group S, accounting for 15.6% of the total number of *bZIP* genes. Among the intron containing genes, the number of introns in open reading frames varied from 1 to 18 and its intron number had a considerable variation among the different GmbZIP groups. For example, subfamilies A, B, C, E, F, H, K and I had 1–6 introns, whereas subfamilies D, G and J contained 7–18 introns, except for GmbZIP71, 113, 123, 101, 110 and 121. We thus propose that both exon loss and gain occurred during the evolution of the *GmbZIP* gene family.

**Fig. 5:**
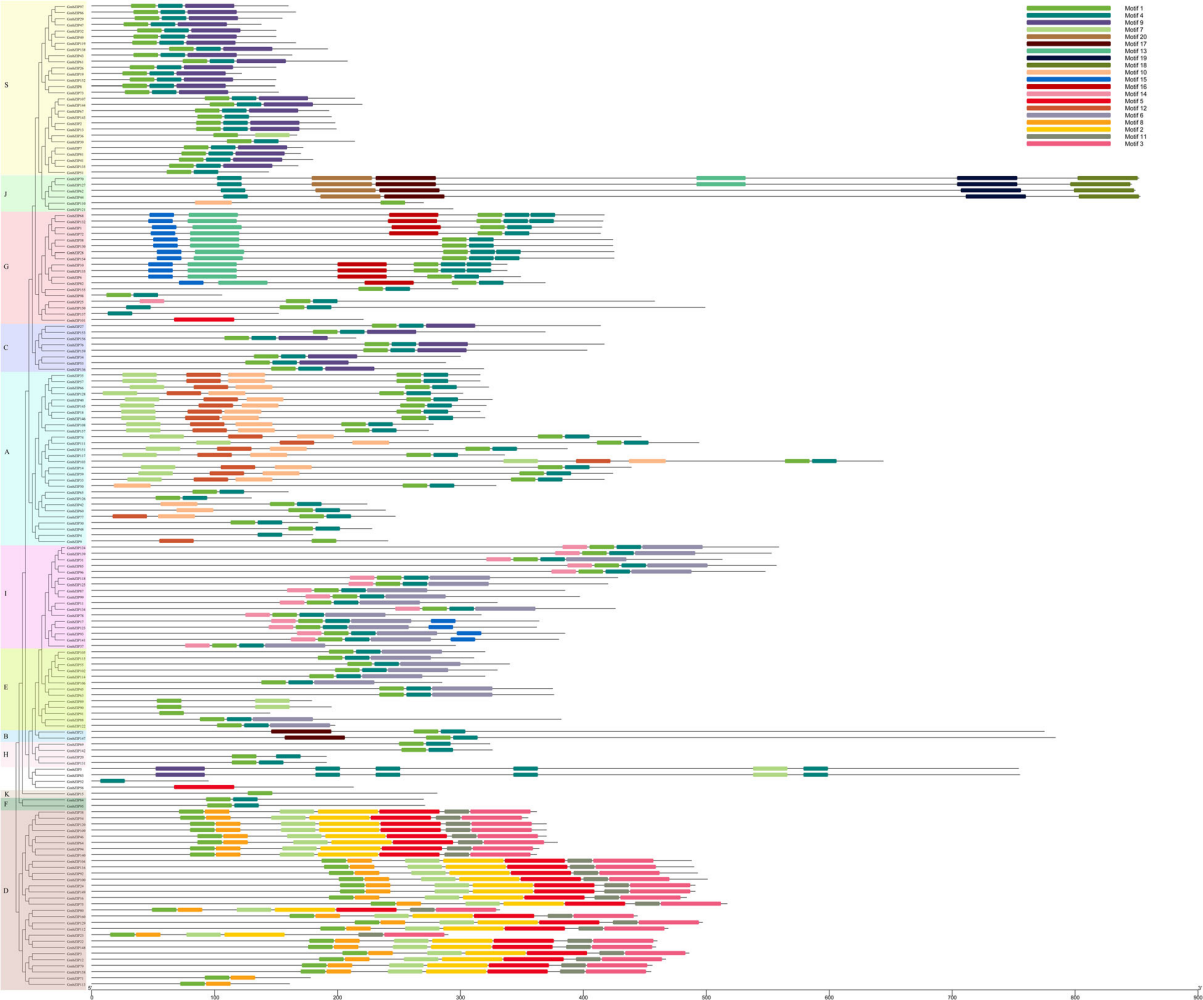
Conserved motifs of soybean bZIP proteins according to the evolutionary relationship. The conserved motifs in the GmbZIP proteins were identified with MEME software. Grey lines denote the non-conserved sequences, and each motif is indicated by a colored box numbered at the top. The length of motifs in each protein was presented proportionally

To obtain insight into the divergence and function of GmbZIP proteins, a total of 20 conserved motifs in the GmbZIPs were captured by the MEME software (Additional file 7: Figure S1) [34]. Most of the GmbZIP proteins within the same subfamily displayed similar motif compositions (Fig. 5), although there was high variance among the different subgroups. For instance, all the subfamilies contain motif1, which was annotated as basic-leucine zipper (bZIP) domain; subgroup A possesses motifs 1, 4, 7, 10 and 12, but different subgroup A members have diversity motifs; subfamily B shares motifs 1, 4 and 17; subfamily C harbors motifs 1, 4 and 9; subfamily D shows motifs 1, 2, 3, 5, 7, 8, and 11; subfamily E has motifs 1, 4 and 6; motifs 1, 4, 13, 15 and 16 appear in the subfamily G; motifs1, 4, 6, and 14 present in the subfamily I; motifs 4, 17, 18, 19 and 20 appear in the subfamily J; motifs 1, 4 and 9 appear in the subfamily S; all the members of subfamilies F and H have motif1 and 4; subfamily K only owns motif1.

### Evolutionary and phylogenetic relationship of soybean and *Arabidopsis* bZIP TFs

A comparison of the genomes of different organisms can be an effective mean to deduce the evolutionary history, origin, and function of uncharacterized genes [35]. Since *Arabidopsis* is one of the best-studied model plant species and the functions of several *Arabidopsis* bZIP genes have been well characterized, we generated a comparative bZIP synteny map of the soybean and *Arabidopsis* genomes. Large-scale syntenies identified 40 *Arabidopsis* genes and 83 soybean genes were orthologous (Fig.4 and Additional file 2: Table S4). Among them, three pairs of syntenic orthologous genes (one to one) were identified: *GmbZIP33*-*AtbZIP15*, *GmbZIP134*-*AtbZIP51*and *GmbZIP112-AtbZIP45*. Therefore, we proposed that these genes are derived from the same ancestor of *Arabidopsis* and soybean. We also identified that one soybean gene corresponds to multiple *Arabidopsis* genes, such as: *GmbZIP117-AtbZIP39/AtbZIP67* and *GmbZIP135*- *AtbZIP5*/*AtbZIP6*. There are also syntenic orthologous gene pairs with one *Arabidopsis* gene corresponding to multiple soybean genes, such as: *AtbZIP17*-*GmbZIP147/GmbZIP21*, *AtbZIP30-GmbZIP31*/*GmbZIP96*, *AtbZIP18*-*GmbZIP137*/*GmbZIP123*/*GmbZIP17*. There are also gene pairs with two *Arabidopsis* genes correspond to the same four soybean genes: *AtbZIP5*/*AtbZIP6*-*GmbZIP135/GmbZIP7*/ *GmbZIP81*/*GmbZIP4 and AtbZIP54*/*AtbZIP55*- *GmbZIP130*/*GmbZIP154*/*GmbZIP28*/*GmbZIP58*. Numbers of synteny events suggested that many *bZIP* genes arose before the divergence of the Arabidopsis and soybean lineages. For further evolutionary studies, Ka and Ks values can be used to predict the selective pressure on duplicated genes, such that Ka/Ks < 1 indicates negative selection, Ka/Ks = 1 indicates neutral selection, and Ka/Ks ratio > 1 indicates positive selection [36]. To explore the divergence of orthologous gene pairs between soybean and *Arabidopsis*, the Ka, Ks and Ka/Ks of the orthologous gene pairs were calculated based on the comparative synteny map. Most of the soybean genes had a Ka/Ks ratio <0.25, with the highest in the *ATbZIP54*-*GmbZIP130*pair (Ka/Ks ratio = 0.35).

**Fig. 6:**
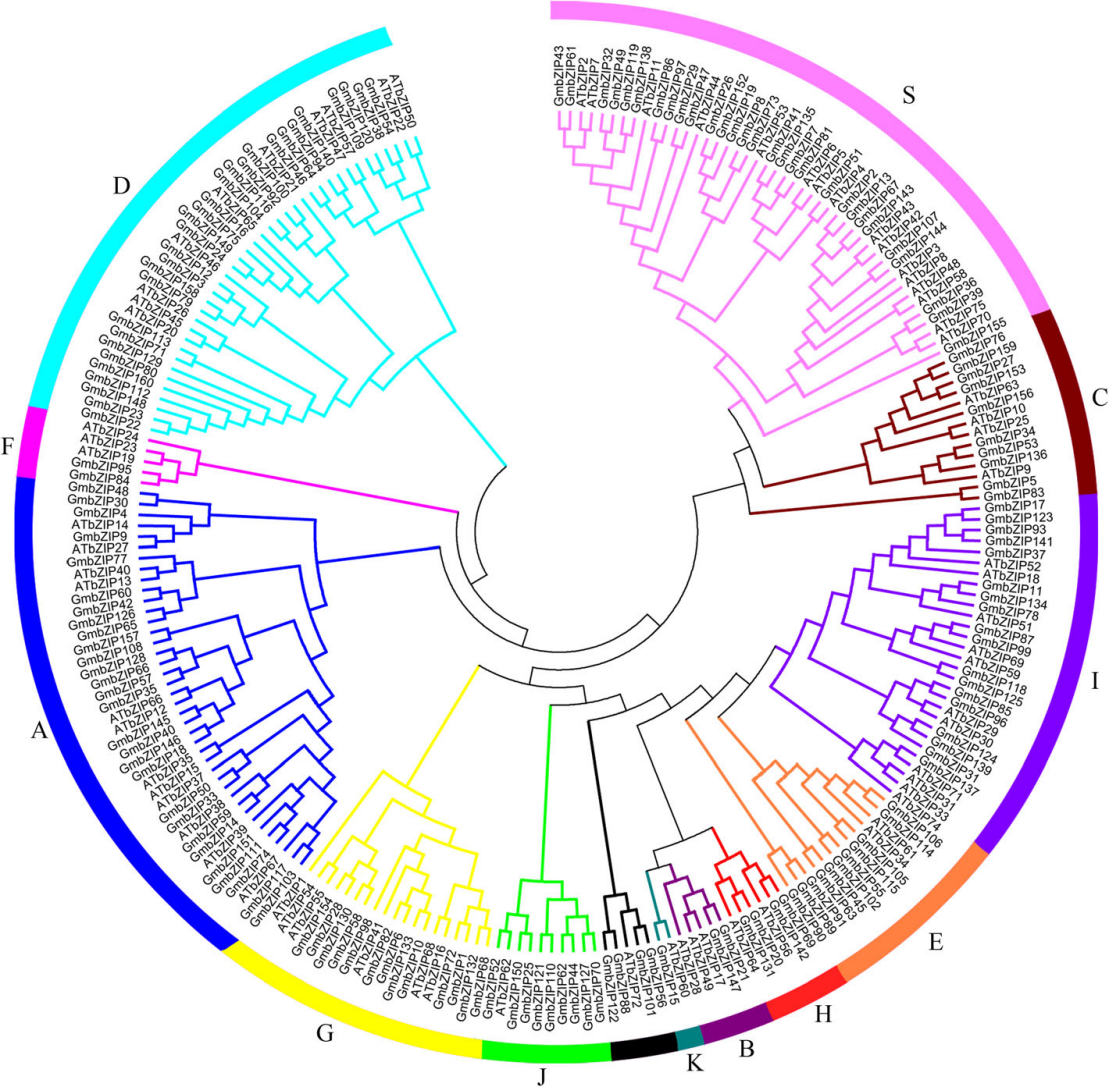
Phylogenetic analysis of soybean and *Arabidopsis* bZIP proteins

In order to further study the evolution of bZIP TFs, an unrooted neighbor-joining (NJ) tree was constructed using the predicted full-length GmbZIP protein sequences and the AtbZIP protein sequences obtained in previous study [37] (Figs. 4 and 6). The grouping in soybean was found to comply with the classification in Arabidopsis, and the *Arabidopsis* group nomenclature (A, B, C, D, E, F, G, H, I, and S) proposed by Jakoby and his colleagues [6] was adopted, along with the two extra groups (J and K) classified by Correa et al. [37]. The result showed that all the GmbZIP proteins were grouped into 12 subfamilies, except for GmbZIP5, GmbZIP83, GmbZIP52 and GmbZIP56. Subfamily A, D and S have nearly 30 GmbZIPs, 28, 29 and 28 respectively; whereas subfamily B, F, H, J and K own less than 6 GmbZIPs, suggesting that the bZIP family differentiated in soybean with diverse functions. Evolutionary analysis also identified that some orthologous bZIPs differentiated between soybean and *Arabidopsis*, indicating that some ancestral bZIP existed prior to the divergence of soybean and *Arabidopsis*.

### Expression profiles of *GmbZIP* genes in different tissues of soybean

**Fig. 7:**
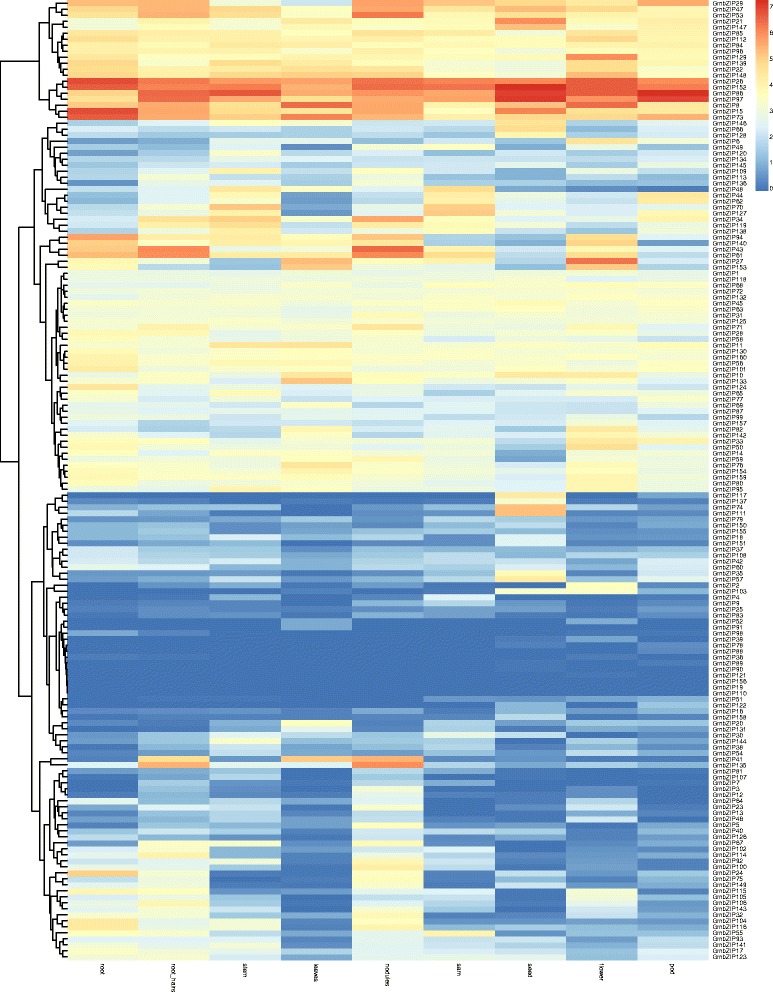
Expression profiles of 160 *GmbZIP* genes in 9 different tissues of soybean. Different colors in map represent gene transcript abundance values as shown in bar at top of figure

To investigate the organ expression pattern of *bZIP* genes in soybean development, we performed transcriptome analysis of soybean tissues at different stages. The result showed that a total of 157 *GmbZIP* genes expressed in different tissues, while the rest of 3*GmbZIP* genes (GmbZIP19, GmbZIP110, GmbZIP156) were not detected in the RNA-seq libraries (Fig. 7, Additional file 3: Table S5). 131 (83.44%) genes exhibited transcript abundance in all tissues, in which 12 genes had high expression levels (value>10). Furthermore, 44 genes show high expression levels in flowers; 28 genes show high expression levels in leaves; 29 genes show high expression levels in pods; 29 genes show high expression levels in seeds; 46 genes show high expression levels in roots; 38 genes show high expression levels in root hair; 38 genes show high expression levels in shoot apical meristem; 37 genes show high expression levels in stem, 49 genes show high expression levels in nodules. Among these genes, some express only in reproductive tissues while some others express only in vegetative tissues. Interestingly, *GmbZIP74* and *GmbZIP111* show high expression level only in seed and barely no expression in vegetative tissues; *GmbZIP6* shows high expression level only in flower; and *GmbZIP109*, *GmbZIP41* and *GmbZIP135* show high expression level in different vegetative tissues while no expression in reproductive tissues.

### Expression profiles of bZIP genes in response to abiotic stress

**Fig. 8:**
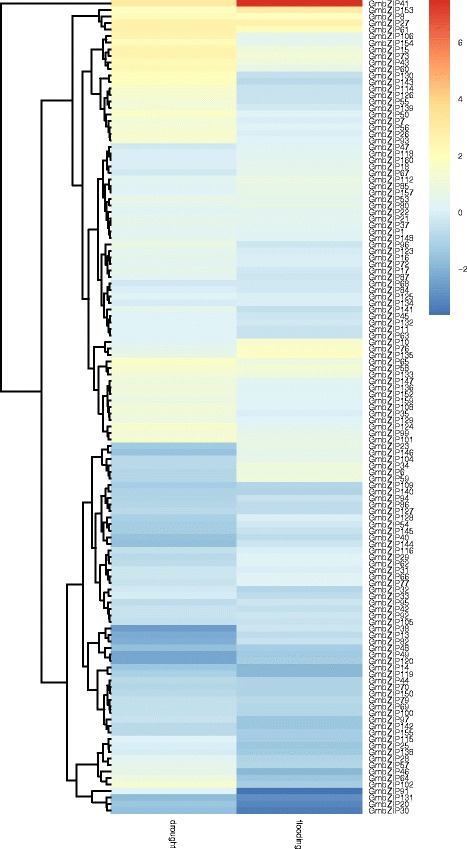
Heat map showing *GmbZIP* expression patterns in soybean under drought and flooding stress. Different colors in map represent gene transcript abundance values after dividing wild type as shown in bar at top of figure

To date, many *bZIP* genes in response to abiotic stress have been identified; some *bZIP* genes were induced by drought, such as in sheep grass (*Leymuschinensis*), apple and rice [7, 38, 39], while some *bZIP* genes were induced by flooding in soybean and Taxodium [40, 41]. To investigate the roles of *bZIP* genes in soybean in response to abiotic stress, an expression analysis of these genes against drought and flood were displayed with the RNA-seq data obtained from Chen et al. [30] (Additional file 8: Table S6 and Additional file 9: Table S7). According to the transcriptome data, 124 out of 160 *GmbZIP* genes were involved in drought stress response. 21*GmbZIP* genes were downregulated significantly (|log2(fold change)|>1), while 31*GmbZIP* genes were upregulated significantly in drought stress. Genes downregulated after drought treatment mainly distribute in the subfamilies A, D and S, while upregulated in subfamilies A, E, G, I and S. 122 out of 160 *GmbZIP* genes were involved in flooding stress response. 19 *GmbZIP* genes showed downregulated significantly; 10 *GmbZIP* genes behaved upregulated significantly in flooding (Fig. 8). Genes downregulated after flooding treatment mainly distribute in the subfamilies A, D, E, H and S, while upregulated in subfamilies C, G and S (Fig. 8). Expression of many *bZIP* genes is induced by both drought and flooding treatment, such as, *GmbZIP42*,*58*,*153*,*27*,*61*,*73* and *8* are upregulated significantly in both abiotic treatment, and *GmbZIP49*, *131*, *30*, *120*, *20*, *119*, *14* and *48* are downregulated significantly. *GmbZIP102* is specifically upregulated after drought treatment, while downregulated after flooding treatment. To verify the accuracy of these data, quantitative real-time PCR analysis was performed to detect the expression of randomly selected *bZIP* genes following abiotic stress. These selected genes contain four parts: up- and down-regulated after drought and flooding treatments (Additional file 10: Figure S2 and Additional file 11: Figure S3). The results are consistent with the gene profiling data.

### Identification of *cis-elements* in *GmbZIP* promoters

To explore the mechanisms of *GmbZIP* response to the abiotic stresses, we searched for 16 stress-related *cis*-elements, such as W-box [42], HSE [43], GARE [44] in the *GmbZIP* promoters. The result showed that more than two *cis*-elements were located in the promoters of all *GmbZIP* genes with the least two *cis*-elements in the promoters of GmbZIP11, 51, 69, 85 and 141 and the most ten in the promoter of GmbZIP121. About 60% (93 out of 160) GmbZIP promoters contain both HSE and MBS (Additional file 12: Table S8).

## Discussion

Soybean, a major legume crop native to east Asia, presents a wealth of resources for utilization, including oils, proteins, mineral nutrients, and natural products such as isoflavonoids that impact human health and nutrition [45]. Compared to other important crops, research progress on soybean has developed slowly, particularly with the aspect of the responses to biotic and abiotic stresses. The *bZIP* family, one of the most important transcription factor family, has been reported to participate in diverse biological processes, such as plants development, organ differentiation, as well as response to environmental stresses. Although the *bZIP* family has been identified in many plant species, the genome-wide identification of *GmbZIPs* has not yet been reported.

In this study, 160 *bZIP* family genes were identified in the soybean genome. Previous studies have identified 75 *bZIPs* in *Arabidopsis* [6], 77 in cassava [3], 85 in rice [46], 64 in cucumber [22], 125 in maize [5]. These data suggested that bZIP in soybean had expanded compared to that in *Arabidopsis*, cassava, rice, cucumber, and maize. Evolutionary analysis indicated soybean *bZIP* family could be divided into 12 subgroups based on cluster analysis and a comparison with Arabidopsis *bZIP* genes. The phylogenetic analysis was also supported by gene structure and conserved motif analyses. Gene structure analysis showed that *GmbZIPs* possess intron with number varying from 0 to 12, and each subfamily displayed similar intron-exon organizations (Fig. 3). We determined that about 15% of the genes had no introns, which is similar to the status in apple, rice and sorghum [21, 23]. We found that the two genes in a duplicated gene pair tended to be clustered into one group, when phylogenetic analysis was performed, such that segmental pairs *GmbZIP17-GmbZIP123* (3introns), *GmbZIP21-GmbZIP147* (1introns) and *GmbZIP119-GmbZIP138* (no intron) were clustered to group I, B and S, respectively. This was also true for the tandem duplicated pairs. Some duplicated genes had the same number of exons and nearly identical lengths, indicating that segmental or tandem duplication expanded the bZIP family. The members of subfamilies D and G harbor more than 8 introns, while other subfamilies present fewer introns. According to a recent study in rice, the rate of intron loss is faster than the rate of intron gain after segmental duplication [46]. Thus, it can be deduced that the subfamilies G and D might contain the original genes. Exon/intron gain/loss is one of mechanism for diversification of multiple gene families [47], and was also observed in this study. For example, *GmbZIP92* contained 12 exons, while the paralogous gene, *GmbZIP103*, had 11 (Fig. 3), indicating a loss of a exons during evolution, a similar pattern is reported for the apple bZIP family [39]. These gain/losses may be the results of chromosomal rearrangements and fusions, and can potentially lead to the generation of functionally distinct paralogs [48]. Conserved motif analysis indicated that all the *GmbZIPs* shared the typical bZIP domain, and each subgroup harbored similar motifs. We found motif 17 and 20 share the same domain, function in phosphatidylcholine protein transfer, so does the motif 18 and 19, which function in signal transduction, and they are very conserved, only contained in the subfamily J, so the subfamily J may be also conserved and functional (Additional file 7: Figure S1). Gene structure and conserved motif analyses showed that the same subfamily harbored similar intron-exon organizations and conserved motifs, indicating the relationship of *GmbZIPs* in the same group was closer during the gene evolution process [49].

Comparative genomics relies on the structuring of genomes into syntenic blocks that display conserved features across the genomes [50]. The synteny analysis denotes functional and evolutionary connections between soybean and *Arabidopsis* syntenic genes [51]. Many *Arabidopsis* bZIP genes response to biotic and abiotic stresses. For example, *AtbZIP1* can mediate sugar signaling, and when carbon nutrients are limited, gain or loss of function of *AtbZIP1* causes changes in the rates of early seedling establishment [52]. *AtbZIP34* is required for Arabidopsis pollen wall patterning and can regulate lipid metabolism and cellular transportin developing pollen [53]. In this study, both syntenic and phylogenetic analyses were performed to assess the relationship to the model plant, *Arabidopsis*, and to infer the functions of orthologous genes. A total of 40 *Arabidopsis bZIP* genes and 83 soybean *bZIP* genes were identified as orthologs. Among these genes, three single orthologous gene pairs between soybean and *Arabidopsis* were identified, indicating that these genes might present in the genome of the last common ancestor of the two species. The remaining gene combinations showed a more severe complex relationship, with many pairs of single *Arabidopsis* genes corresponding to multiple soybean genes and single soybean genes corresponding to multiple *Arabidopsis* genes. Since some of the *bZIP* genes present in these two species that could not be mapped into any syntenic blocks, this may be explained by the fact that after the speciation of soybean and *Arabidopsis*, their genomes have experienced multiple rounds of important chromosomal rearrangement and fusions, followed by selective gene loss, which can obscure the identification of chromosomal syntenies [54, 55]. In general, we deduce that some of the soybean genes seemed to share a common ancestor with their *Arabidopsis bZIP* counterparts, and since all the Ka, Ks, and Ka/Ks of the syntenic orthologous gene-pairs had Ka/Ks ratios < 1, they might undergo a strong Darwinian purifying positive selection.

In plants, basic region/leucine zipper motif (bZIP) transcription factors regulate processes including pathogen defense, light and stress signaling, seed maturation and flower development. The function of some bZIP genes in soybean has been reported, for instance, overexpression of *GmbZIP110* can improve the tolerance of salt [56]. *GmbZIP44*, *GmbZIP62* and*GmbZIP110* are the negative regulators of ABA signaling [57]. Based on the expression data (Additional file 8: Table S6), all investigated soybean organs expressed at least one *bZIP* gene, indicating that they may play a broad role in soybean development. Most of the expressed genes were detected to some degree in roots, stems, leaves, as well as in flowers and seeds, which is consistent with previous studies in *Arabidopsis* [6]. In this study, no less than 75.6% *bZIP* genes displayed transcriptional changes after abiotic stress treatment, including drought and flooding (Fig. 8 and Additional file 3: Table S5 and Additional file 8: Table S6). Gene expression analysis has showed that more genes changed after drought treatment than flooding, so the bZIP family may play more dominantly role in regulating drought stress. Collectively, these findings indicated that *bZIP* genes might contribute to the robust resistance to water stress and provide a foundation for further research on the *bZIP*-mediated water stress response in soybean. Some *GmbZIP* genes were not detected in the database, and the similar circumstance was observed in apple and grape [39, 58], we therefore hypothesize that these genes may have been silenced or their expression is too weak to be detected. In our study, most of *GmbZIP* genes were responsive to drought and flood, and consistent with expression, their promoters contained at least one out of three drought-relative *cis*-elements (ABRE, MBS and W-box).

## Conclusions

In general, we performed the first genome-wide identification of the soybean bZIP TF family and conducted a detailed investigation of their structure and organization. We also characterized the expression of some *GmbZIP* genes following various abiotic stresses. These results may prove useful in developing strategies for the future improvement of stress tolerance in soybean.

## Additional files

Additional file 1: **TableS3.** Synteny blocks of bZIP genes within soybean genome. (XLSX 19 kb)

Additional file 2: **Table S4.** Synteny blocks of bZIP genes between soybean and *Arabidopsis* genomes. (XLSX 19 kb)

Additional file 3: **Table S5.** The expression profiles of the soybean *bZIP* genes in different tissues. (XLSX 5886 kb)

Additional file 4: **Table S9.** Primers used in qRT-PCR analysis. (XLSX 10 kb)

Additional file 5: **Table S1.** Characteristics of bZIPs in soybean. (XLSX 17 kb)

Additional file 6: **Table S2.** The accession numbers of bZIPs in soybean and *Arabidopsis*. (XLSX 5633 kb)

Additional file 7: **Figure S1.** 20 conserved motifs and functions of some motifs. (DOCX 720 kb)

Additional file 8: **Table S6.** The expression profiles (log2-based values) of the soybean bZIP genes after drought treatment. In this experiment, two-week-old soybean seedlings were transferred to the drought conditions for a week, and drought stress was imposed by withholding water for 7 days. After 7 days of treatment, the leaves were collected for experiment [30]. (XLSX 18 kb)

Additional file 9: **Table S7.** The expression profiles (log2-based values) of the soybean bZIP genes after flooding treatment. In this experiment, two-week-old soybean seedlings were transferred to the flooding conditions for a week, and flooding stress was imposed by placing the pots into a bigger pot with a trashcan liner filled up to a water level of 4 cm above the soil surface for 7 days. After 7 days of treatment, the leaves were collected for experiment [30]. (XLSX 16 kb)

Additional file 10: **Figure S2.** Validation of drought treatment RNA-seq data. (A) RNA-seq data of downregulated genes after drought treatment. (B) Validation of down regulated genes among drought sequencing data by real-time quantitative PCR. (C) RNA-seq data of upregulated genes after drought treatment. (D) Validation of up regulated genes among drought sequencing data by real-time quantitative PCR. FPKM represents Fragments Per Kilobase per Million fragments according to previously described [59]. (TIFF 203 kb)

Additional file 11: **Figure S3.** Validation of flooding treatment RNA-seq data. (A) RNA-seq data of downregulated genes after flooding treatment. (B) Validation of down regulated genes among flooding sequencing data by real-time quantitative PCR. (C) RNA-seq data of upregulated genes after flooding treatment. (D) Validation of up regulated genes among flooding sequencing data by real-time quantitative PCR. (TIFF 209 kb)

Additional file 12: **Table S8.** Cis-elements in *GmbZIP* promoters. (XLSX 20 kb)

## Abbreviations

ABA: Abscisic acid
At: *Arabidopsis thaliana*
bZIP: Basic Leucine Zipper
Gm: *Glycine max*
GSDS: Gene Structure Display Server
HMM: Hidden Markov Models
MEGA: Molecular Evolutionary Genetics Analysis
MEME: Multiple Em for Motif Elicitation
NJ: Neighbor-joining
Os: *Oryza sativa*
qRT-PCR: Quantitative real-time PCR
RNA-seq: RNA sequencing
SMART: Simple Modular Architecture Research Tool
TF: Transcription factor
Zm: *Zea mays*
