# Supplementary material for: Evolutionary and expression analyses of soybean basic Leucine zipper transcription factor family
Source: BMC Genomics. 2018 Feb 22;19:159. doi: 10.1186/s12864-018-4511-6 (PMC5824455; doi:10.1186/s12864-018-4511-6)
Supplement: Supplementary file 7 — Figure S1. 20 conserved motifs and functions of some motifs. (DOCX 720 kb) [file 12864_2018_4511_MOESM7_ESM.docx]

| **Figure S1. 20 conserved motifs and functions** |
| --- |
| 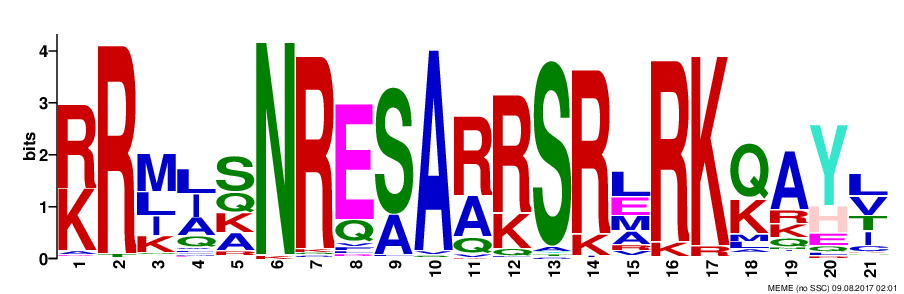 |
| motif1: basic region leucin zipper. |
| 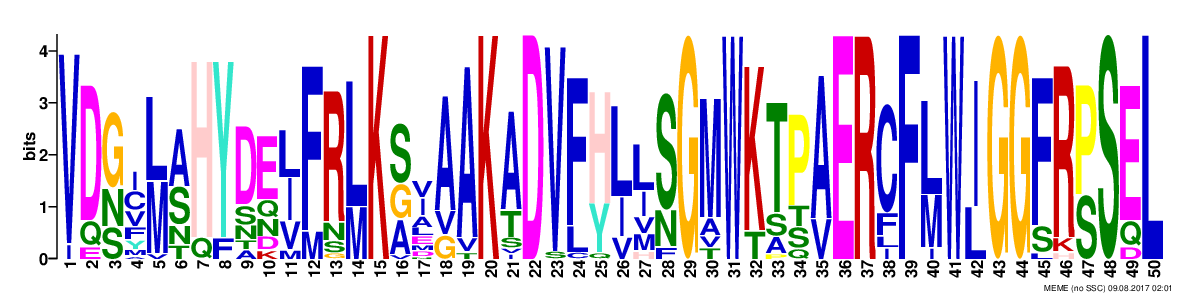 |
| motif2: sequence-specific DNA binding. |
| 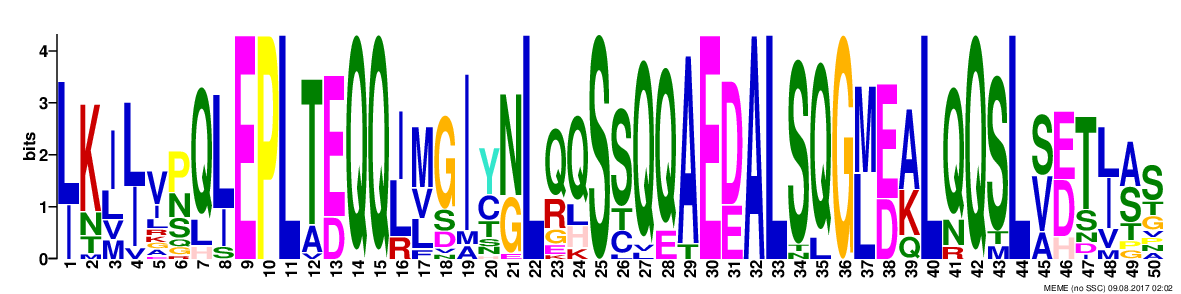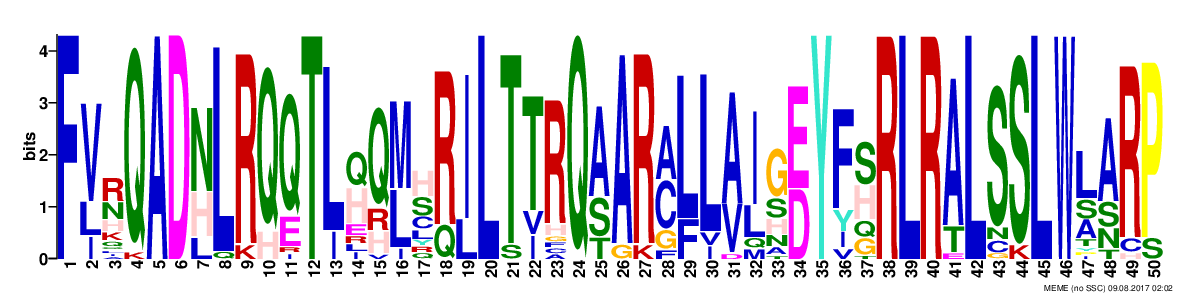 |
| motif3 |
| 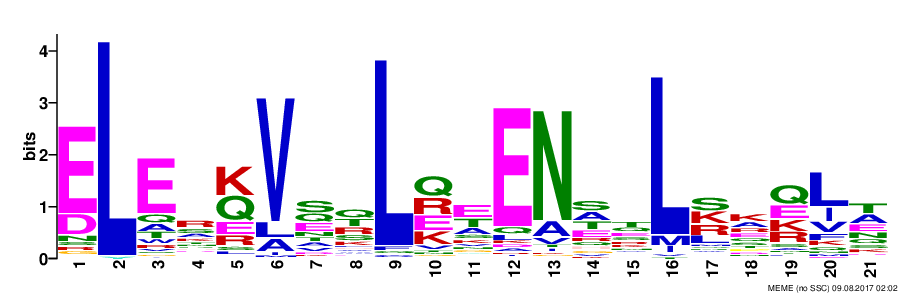 |
| motif4: BRLZ, basic region leucin zipper; sequence-specific DNA binding transcription factor activity. |
| 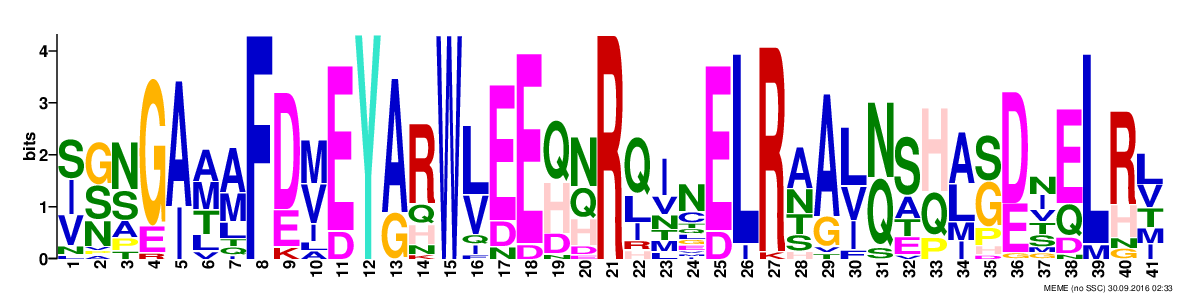 |
| motif5 |
| 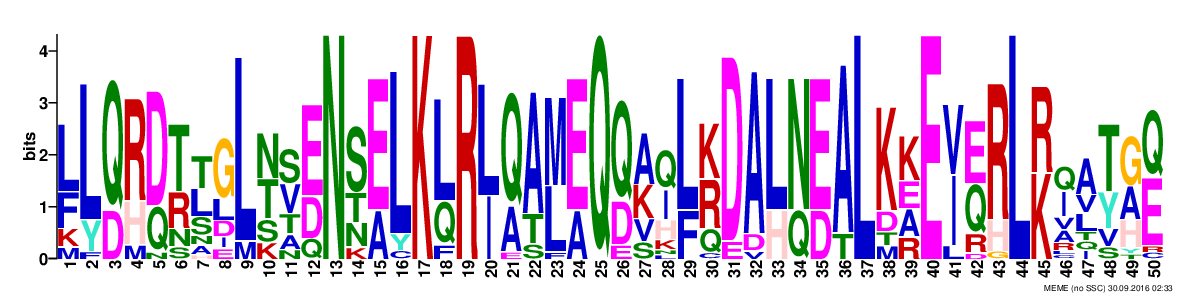 |
| motif6 |
| 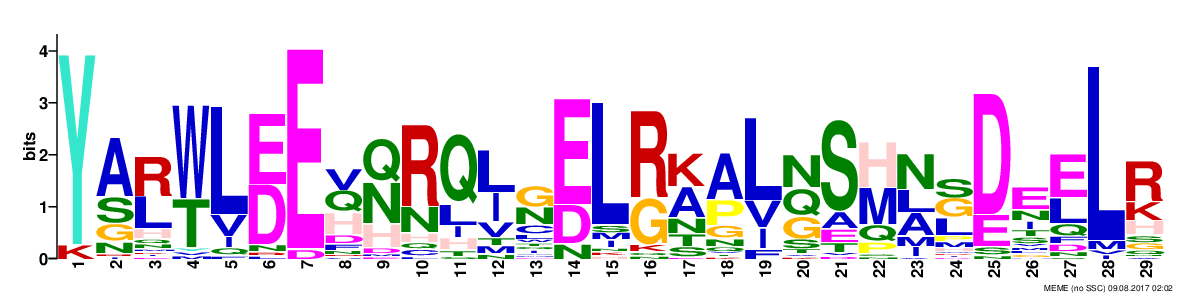 |
| motif7 |
| 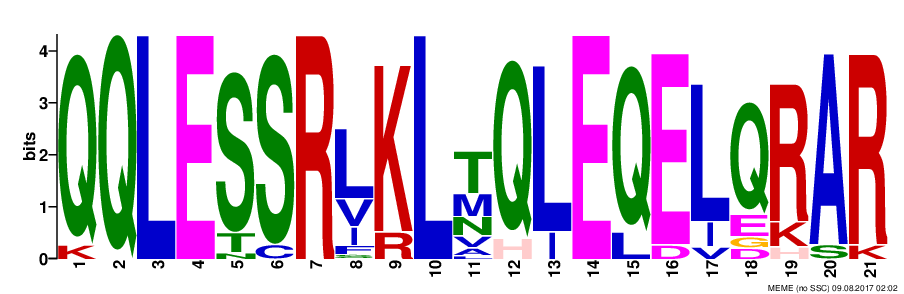 |
| motif8 |
| 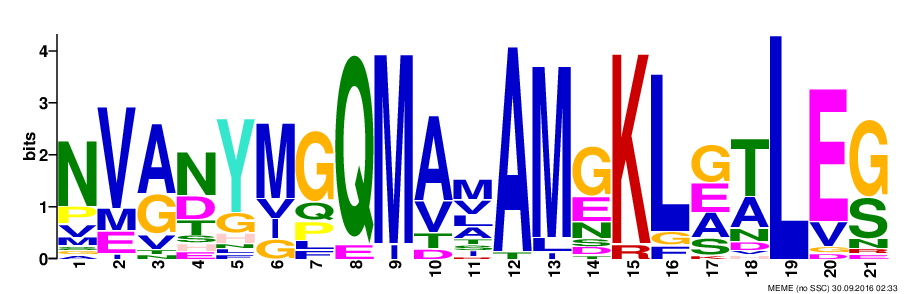 |
| motif9: bZIP_C, basic region leucin zipper |
| 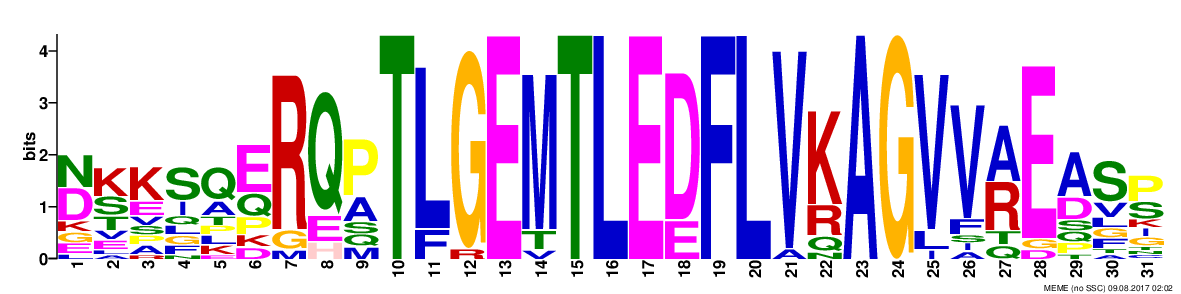 |
| motif10 |
| 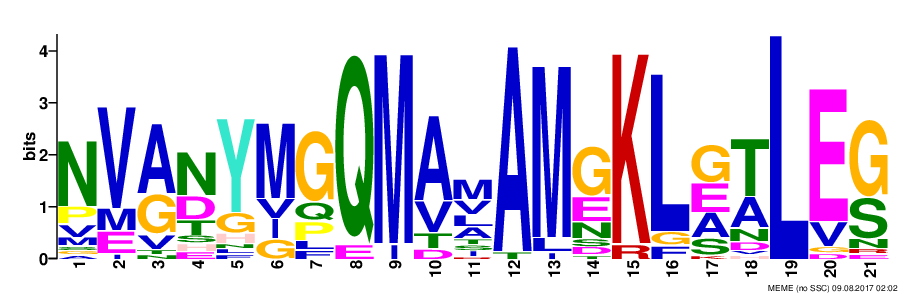 |
| motif11 |
| 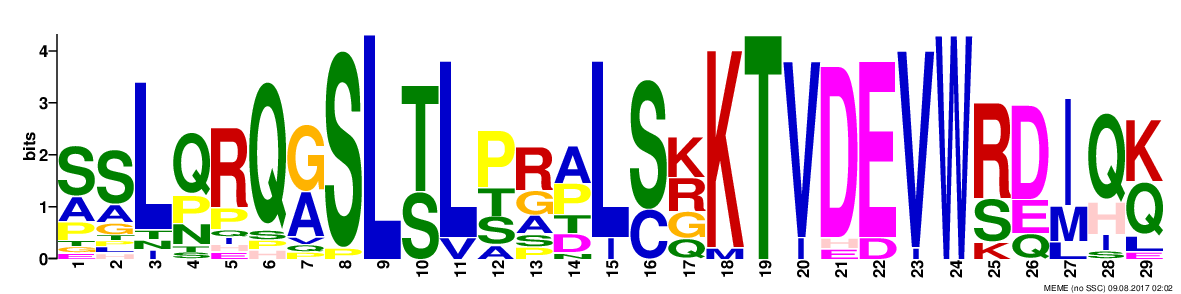 |
| motif12 |
| 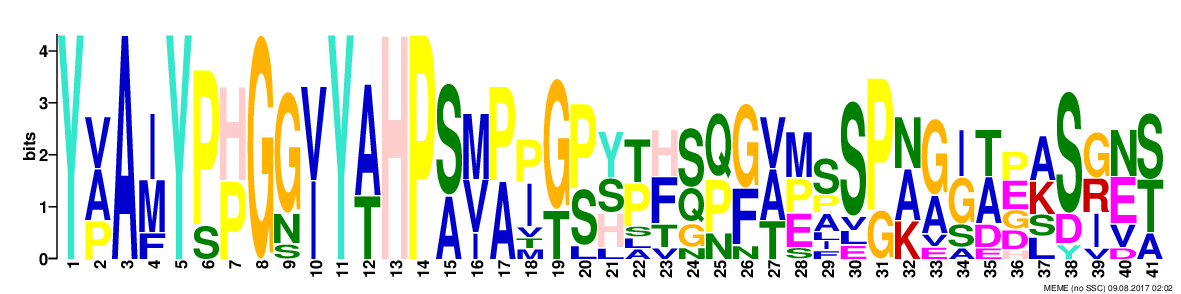 |
| motif13 |
| 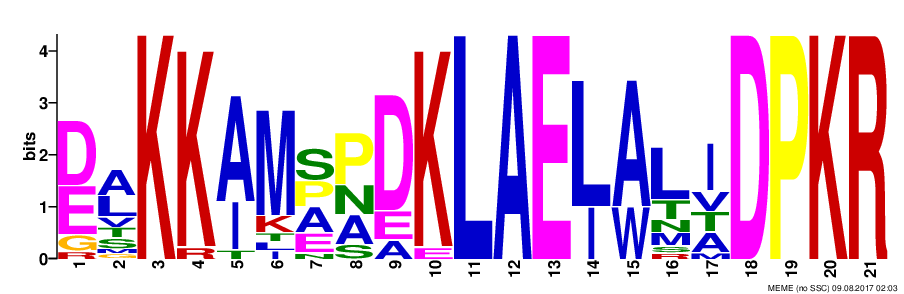 |
| motif14 |
| 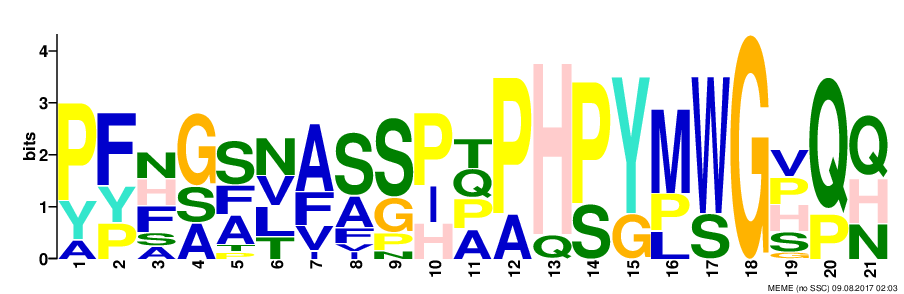 |
| motif15 |
| 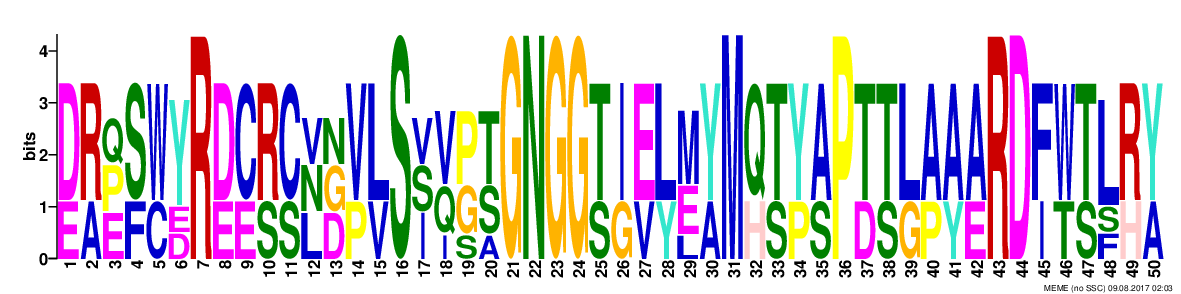 |
| motif16 |
| 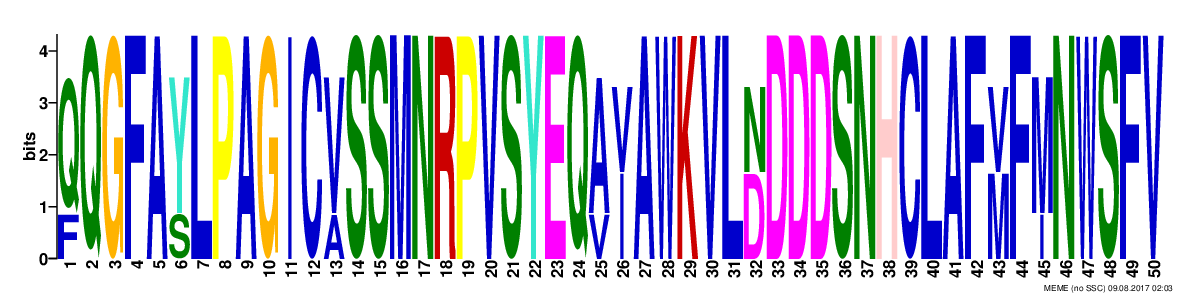 |
| motif17: putative lipid-binding domain in StAR and phosphatidylcholine transfer protein. |
| 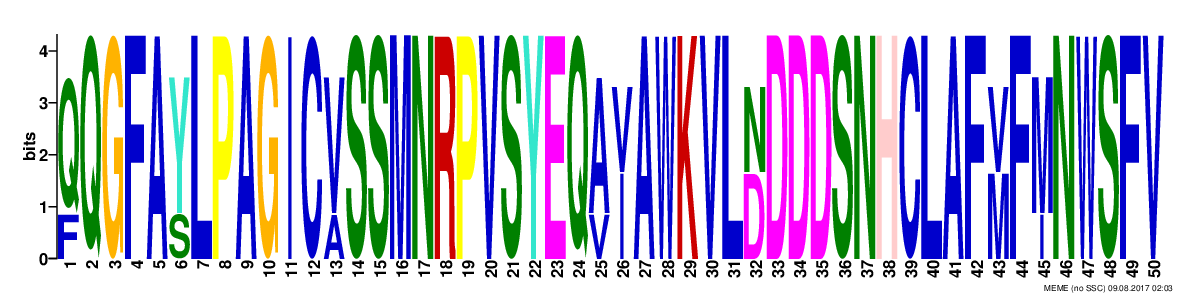 |
| motif18: The MEKHLA domain shares similarity with the PAS domain and is found in the 3' end of plant HD-ZIP III homeobox genes, and bacterial proteins. |
| 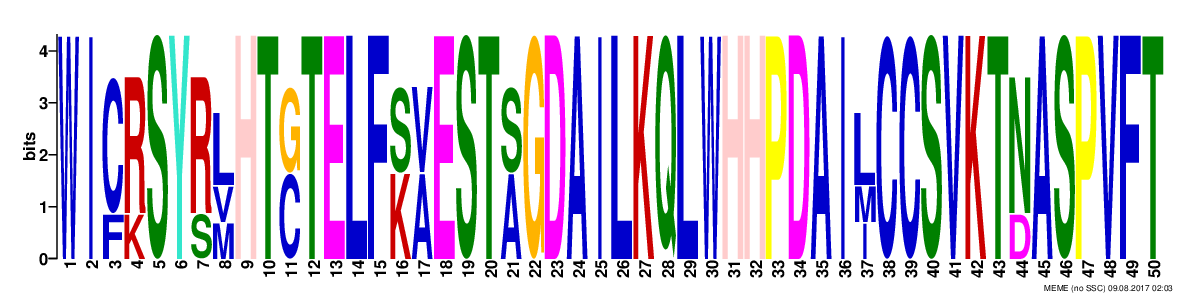 |
| motif19: The MEKHLA domain shares similarity with the PAS domain and is found in the 3' end of plant HD-ZIP III homeobox genes, and bacterial proteins. |
| 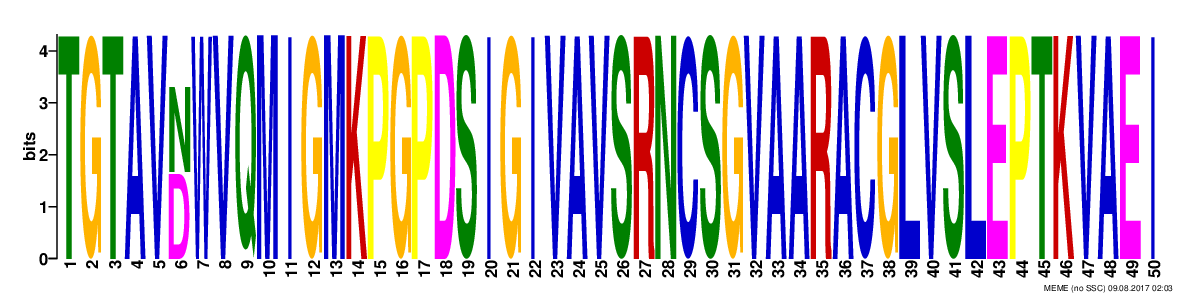 |
| motif20: putative lipid-binding domain in StAR and phosphatidylcholine transfer protein |
