# Supplementary figures and images for: Evolutionary and expression analyses of soybean basic Leucine zipper transcription factor family
Source: BMC Genomics. 2018 Feb 22;19:159. doi: 10.1186/s12864-018-4511-6 (PMC5824455; doi:10.1186/s12864-018-4511-6)

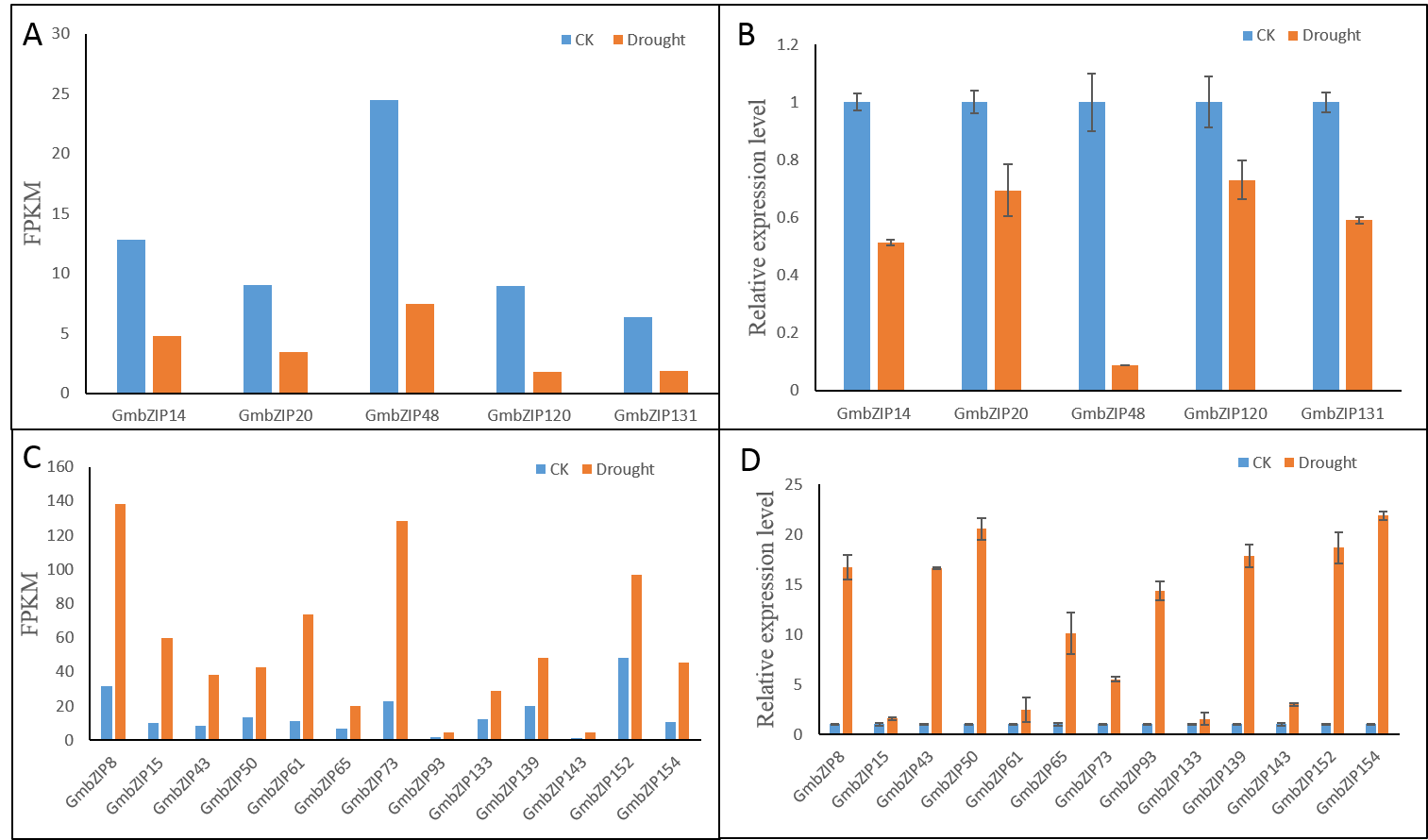

Supplement: Supplementary file 10 — Figure S2. Validation of drought treatment RNA-seq data. (A) RNA-seq data of downregulated genes after drought treatment. (B) Validation of down regulated genes among drought sequencing data by real-time quantitative PCR. (C) RNA-seq data of upregulated genes after drought treatment. (D) Validation of up regulated genes among drought sequencing data by real-time quantitative PCR. FPKM represents Fragments Per Kilobase per Million fragments according to previously described [59]. (TIFF 203 kb) [file 12864_2018_4511_MOESM10_ESM.tif]

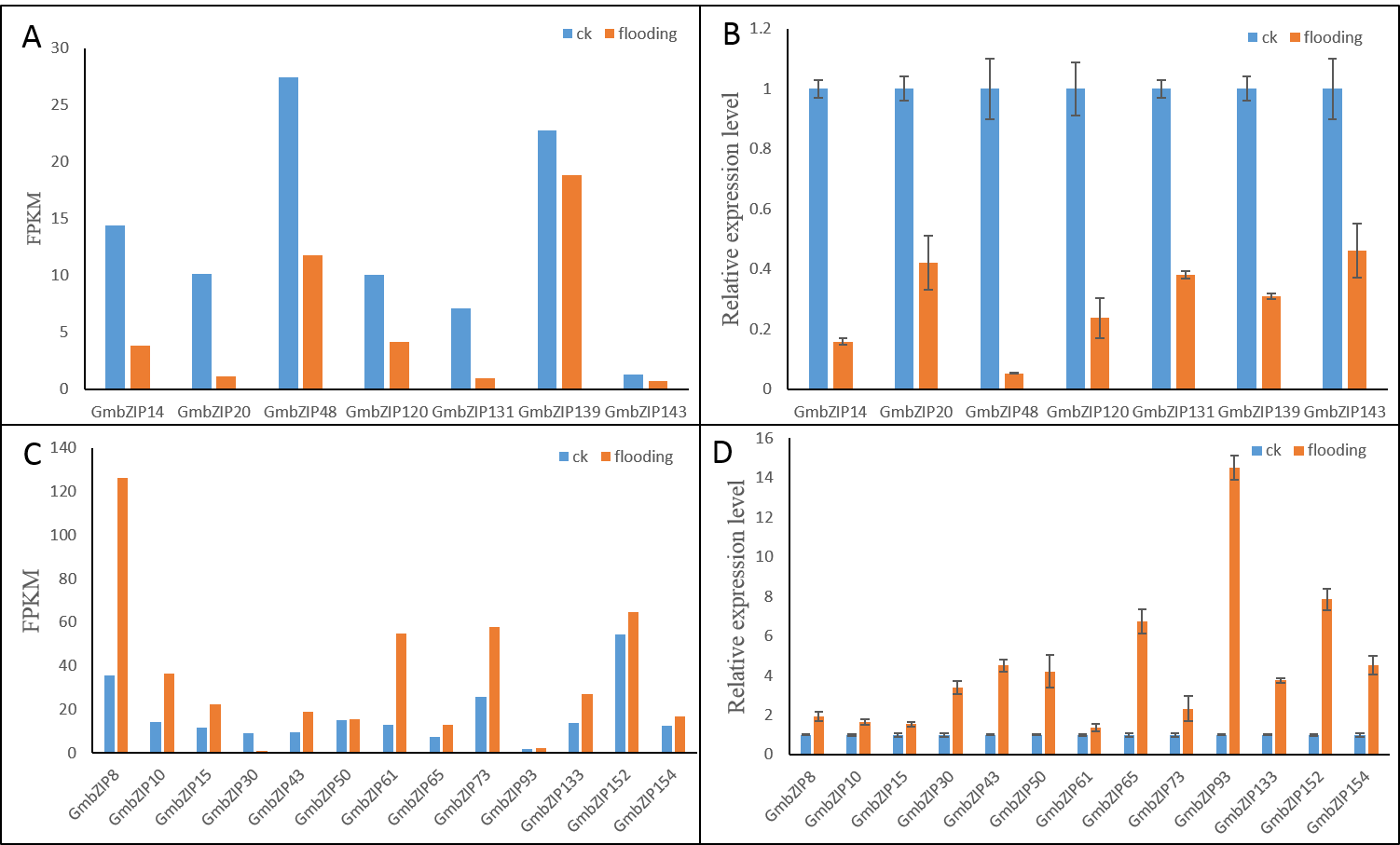

Supplement: Supplementary file 11 — Figure S3. Validation of flooding treatment RNA-seq data. (A) RNA-seq data of downregulated genes after flooding treatment. (B) Validation of down regulated genes among flooding sequencing data by real-time quantitative PCR. (C) RNA-seq data of upregulated genes after flooding treatment. (D) Validation of up regulated genes among flooding sequencing data by real-time quantitative PCR. (TIFF 209 kb) [file 12864_2018_4511_MOESM11_ESM.tif]
